# The relationship of out-of-pocket expenses to quality of life and disease coping in breast cancer patients receiving chemotherapy

**DOI:** 10.1590/1806-9282.20241349

**Published:** 2025-03-31

**Authors:** Yasemin Özyer Güvener, Zeliha Koç

**Affiliations:** 1 Sinop University, Faculty of Health Science – Sinop, Turkey.; 2 Ondokuz Mayıs University, Faculty of Health Science – Samsun, Turkey.

**Keywords:** Breast cancer, Chemotherapy, Out-of-pocket expenditures, Quality of life, Coping, Adaptation

## Abstract

**OBJECTIVE:**

This study was carried out to determine the relationship of out-of-pocket expenses to quality of life and disease coping in breast cancer patients receiving outpatient treatment in a chemotherapy unit.

**METHODS:**

This was a descriptive and correlational survey study. The sample of the study consisted of 322 breast cancer patients. An embedded mixed design, in which quantitative and qualitative research methods are employed together, was used in the research. Quantitative data were collected using the Functional Assessment of Cancer Therapy-Breast and the Coping and Adaptation Processing Scale.

**RESULTS:**

After the qualitative interviews held with the patients, it was determined that the diagnosis and treatment of cancer affected the patients physically, mentally, socially, and economically, they spent out-of-pocket, and these expenses affected their quality of life.

**CONCLUSION:**

It can be said that the out-of-pocket expenses of breast cancer patients during the chemotherapy treatment affected their quality of life, disease coping, and adaptation.

## INTRODUCTION

Cancer is a disease with very high mortality and morbidity rates. According to the GLOBOCAN 2018 data, 18.1 million people were diagnosed with cancer and 9.6 million people died due to cancer. When compared to other cancers, the prevalence of breast cancer is relatively high in both developed and developing countries: 2,088,849 women were diagnosed with breast cancer, and 626,679 women died due to breast cancer in 2018^1^. In Turkey, the age-standardized cancer rate was determined as 259.9 per 100,000 in men and 183.2 per 100,000 in women^2^. In 2020, 233,834 people were diagnosed with cancer in Turkey and 24,175 of these cases were breast cancer patients. Moreover, in the same year, 126,335 people died due to cancer and 7,161 of these were breast cancer patients^3^.

### Out-of-pocket expenses

Cancer is a disease that affects patients and their relatives in many ways. High medical expenses related to cancer and disease-related changes in employment status can affect the economic situation of cancer patients and their relatives^4^. Family members may have to take time off work in order to provide support and care to patients, not remorate the course of treatment, and to meet the emotional needs of patients^5^.

Out-of-pocket health expenses are payments made by patients or households. The most distinctive feature of out-of-pocket expenses is that some or all of them cannot be recovered from another person or institution as a repayment or other form of aid^6,7^. The presence or absence of health insurance and the income level of the household can affect out-of-pocket health expenses. The financial burden of out-of-pocket health expenses on households may vary depending on the income level^6,8,9^.

### Quality of life

Quality of life is a subjective assessment of one's physical, psychological, and social well-being^10^. Quality of life refers to the assessment of each patient regarding their current level of functionality and their satisfaction with it^11^. Moreover, it is important to evaluate the quality of life of oncology patients since this is used to evaluate the quality of care and treatment outcomes^10,11^.

Health expenses can have a direct impact on the quality of life and life expectancy of individuals^12^. For this reason, it is important to determine the out-of-pocket expenses of individuals and the contribution of the services to these costs in order to prevent oncology patients from having out-of-pocket health expenses and to improve their quality of life^13,14^.

### Coping and adaptation

In addition to oncology patients’ quality of life, the personal characteristics of individuals and how they cope with the disease are also important^15^. The excessive financial burden of cancer can harm the physical and emotional health of patients and their relatives, forcing them to develop cost-related coping strategies^16^.

### Objectives

No research in Turkey has determined the relationship of out-of-pocket expenses to quality of life and disease coping in breast cancer patients receiving chemotherapy. This gap constituted the starting point of this research. Appropriate recommendations and strategies will be provided in line with the findings obtained in this research.

### Research questions

- Do breast cancer patients spend out-of-pocket during chemotherapy treatment?
- Do out-of-pocket expenses of breast cancer patients differ according to their sociodemographic and clinical characteristics?
- Is there a relationship between the out-of-pocket expenses of breast cancer patients and their quality of life (FACT-B) and disease-coping status (CAPS)?
- How does chemotherapy treatment affect breast cancer patients’ disease, out-of-pocket expenses, quality of life (FACT-B), and the process of coping with the disease (CAPS)?

## METHODS

This descriptive and correlational study was carried out to determine the relationship of out-of-pocket expenses to quality of life and disease coping in breast cancer patients receiving outpatient treatment in the chemotherapy unit. The research was conducted with breast cancer patients who received outpatient treatment in the chemotherapy unit of Ondokuz Mayıs University Health Application and Research Center between 29 October 2019 and 28 October 2020. In the study, the sample size was determined using the formula used for a known universe. While determining the size of the sample that would represent the universe in the study, a total of 351 breast cancer patients who received outpatient treatment in the chemotherapy unit between August 2017 and August 2018, 1–2 years prior to the study, were taken as reference. The number of samples with a known universe was calculated as 319 with an error of 2% at a confidence interval of 98%. Considering any data loss, the data collection process was terminated when 322 patients were reached.

### Data collection tools

A semi-structured interview form was used to collect the qualitative data for the research. A Patient Information Form, the Functional Assessment of Chronic Therapy Breast Cancer (FACT-B), and the Coping and Adaptation Processing Scale (CAPS) were used to collect the quantitative data for the study.

A semi-structured questionnaire was used during interviews, which were held before or after their chemotherapy sessions, depending on the preference of the patients. Data saturation was reached after interviews with 27 breast cancer patients.

### Patient information form

The form consists of questions to determine the sociodemographic and clinical information.

### Coping and Adaptation Processing Scale

CAPS was developed by Callista Roy to understand and measure the complex, multidimensional, hierarchical structure of coping^17^. The Turkish validity and reliability study of the scale was conducted by Catal^18^. CAPS is generally used to describe the coping and adaptation strategies of individuals in critical and difficult situations. It consists of 47 items and 5 subscales. Catal reported that Cronbach's alpha reliability coefficient of the scale was 0.94 and that Cronbach's alpha reliability coefficients of the subscales ranged from 0.78 to 0.86. In this study, Cronbach's alpha reliability coefficient of the CAPS was determined to be 0.89.

### Functional Assessment of Cancer Therapy-Breast Cancer

The FACT was developed by Cella et al. to target chronic disease management^19^. The validity and reliability studies were performed by Brady et al. and Hahn et al.^20,21^. In this study, the fourth version of the FACT-B, of which the validity and reliability were established by Hahn et al., was used^21^. The FACT-B is a five-point Likert-type scale consisting of 37 items and 5 subscales.

The Cronbach alpha reliability coefficients of the subscales of physical well-being, social/family well-being, emotional well-being, functional well-being, and breast cancer/additional concerns were found as 0.83, 0.76, 0.82, 0.91, and 0.65, respectively. In this study, the Cronbach alpha reliability coefficient of FACT- B was found to be 0.93. The Cronbach alpha reliability coefficients of the subscales of physical well-being, social/family well-being, emotional well-being, functional well-being, and breast cancer/additional concerns were determined to be 0.92, 0.90, 0.63, 0.92, and 0.65, respectively.

### Ethical considerations

The study was initiated after receiving permission from the Clinical Research Ethics Committee of Ondokuz Mayıs University (14.09.2018/ 1885). In order to collect the data, written permission was also obtained from the Health Application and Research Center of Ondokuz Mayıs University (17.10.2018/Nr:15374210-010.06.99-E.115442), and verbal informed consent was obtained from the patients participating in the study. The study was conducted in accordance with the Declaration of Helsinki and the ethical standards of the institution.

### Statistical analyses

The quantitative data obtained in the study were analyzed using the IBM SPSS V23 package program. The fitness to normal distribution was evaluated using the Kolmogorov-Smirnov test. The chi-square test was used to compare categorical variables; the independent paired samples’ t-test was used to compare quantitative variables according to paired groups for normally distributed data; the Mann-Whitney U test, one of the nonparametric tests, was used for comparison of the data without normal distribution. In order to compare three or more groups, one-way ANOVA was used for normally distributed data, and the Kruskal-Wallis test was used for the data without normal distribution. To measure the correlation between quantitative variables, the Pearson coefficient was used for normally distributed data, while Spearman's rho coefficient was used for the data without normal distribution. Statistical significance was taken as p<0.05.

## RESULTS

Socio-demographic and clinical information of the patients is presented: 32.0% were at stage 2, 68.6% received chemotherapy every 21 days, and 14.6% had to quit their job due to chemotherapy (Table 1).

**Table 1:** Distribution of patients’ clinical characteristics (n=322).

Characteristics		n	%
Clinical stage of disease	Stage 1	62	19.3
	Stage 2	103	32.0
	Stage 3	82	25.5
	Stage 4	75	23.2
Frequency of chemotherapy	Once a week	99	30.7
	Once every 21 days	221	68.6
	Once a month	2	0.6
Patient leaving job due to chemotherapy treatment	Yes	48	14.6
	No	274	85.4
Work life of family members affected by chemotherapy treatment	Yes	73	22.7
	No	249	77.3
Utilization of other sources of income by family members while covering treatment expenses	Yes	114	35.4
	No	208	64.6
If yes, sources used by family members to cover the treatment expenses (n=114)	Borrowing money and receiving help from friends/relatives	88	32.8
	Bank loan	26	9.7
Out-of-pocket expenses due to chemotherapy treatment (transportation, food, accommodation, etc.)	Yes	320	99.4
	No	2	0.6
Presence of a companion during chemotherapy treatment	Yes	318	98.7
	No	4	1.3
Out-of-pocket expenses for the companion due to chemotherapy treatment (transportation, food, accommodation, etc.)	Yes	318	98.7
	No	4	1.3

The mean out-of-pocket expenses of the patients in a month were 165.4±187.7 dollars (Table 2).

**Table 2:** Distribution of out-of-pocket expenses of patients in 1 month.

Type of expenses	n	%	Mean±SD	Median (Min–Max)
Expenses for patient's transportation, food, and accommodation during chemotherapy treatment ($)	320	99.4	11.8±9.2	12 (1–48)
Expenses for companion's transportation, food, and accommodation during chemotherapy treatment ($)	318	98.7	12.0±13.0	10 (1–79)
Drug expenses ($)	66	20.4	15.9±1.4	6 (0.5–131)
Supplementary health insurance expenses ($)	18	5.6	72.5±51.6	81 (6–179)
Contributory expenses for private examination ($)	215	66.8	17.3±13.6	14 (11–179)
Expenses for psychiatrist and psychologist ($)	15	4.65	28.9±15.3	30 (6–60)
Private health insurance expenses ($)	8	2.5	19.2±17.6	14 (7–60)
Expenses for medical supplies and equipment (glasses, lenses, breast prostheses, and hearing aids) ($)	55	82.9	49.7±49.7	37 (3–239)
Expenses for medical supplies (batticon, plaster, disinfectant, gauze, mask, thermometer, blood pressure monitor, corset, tourniquet, bandage, etc.) ($)	82	25.5	10.3±13.3	2 (2–60)
Expenses for medical analysis costs ($)	88	27.3	32.9±25.9	24 (5–179)
Expenses for alternative/complementary therapies (phytotherapy, herbal products, and aromatherapy) ($)	29	9.00	53.9±100.3	19 (2–477)
Indirect expenses related to treatment (homemade creams, divination, turbe visits, and visiting a khoja) ($)	20	6.2	40.3±79.2	12 (3–358)
Expenses for side effects (special clothes, wigs, scarves, hats, etc.) ($)	33	10.2	47.6±70.3	24 (3–378)
Expenses for natural/organic products (molasses, propolis, organic vegetables, and fruits, etc.) ($)	102	31.7	20.5±12.8	18 (2–60)
Expenses for supplementary food and supplements (meat, fish, dairy products, etc.) ($)	107	33.2	24.1±20.9	23 (2–179)
Expenses for assisted services (house cleaning, childcare, etc.) ($)	74	22.0	30.0±31.6	20 (9–179)
Mean out-of-pocket expenses per month ($) (Mean±SD) 165.4±187.7				
Mean monthly income loss of patients who left their job ($) (Mean±SD) 180±139.7				
Mean monthly income loss due to the inability of family members to work ($) (Mean±SD) 110.6±96.1				
Mean family income loss ($) (Mean±SD) 157.3±156.26				

Min, minimum; Max, maximum; SD: standard deviation. $1=34.04 Turkish Lira.

Table 3 shows the main themes that were determined as the effects of cancer, out-of-pocket expenses in the care and treatment process, and the effect of out-of-pocket expenses on the quality of life and disease coping and adaptation after the qualitative interviews held with the patients (Table 3).

**Table 3:** Main themes related to the experiences of patients during chemotherapy (n=27).

Categories	Sub-theme	Representative quotes
Cancer effects	Alopecia Fear of reduced fertility İnability to look in the mirror Fatigue, weakness, and pain Social isolation Fear of death Depression Sadness and crying	"When the doctor said I was going to get chemotherapy, I thought about hair loss. That's probably what affected me the most. Rather than the cancer diagnosis, the effects of chemotherapy made me very sad." "…Will my fertility be affected, since chemotherapy drugs interrupt menstruation? I think this is very important for a woman. I panicked when the doctor told me that I could go through the menopause and not have a child."
Out-of-pocket expenses	Financial hardship Borrow money Leaving work Out-of-pocket spending Selling one's property Get a bank loan	"We spend a lot of money to come to chemotherapy. Especially when I had my surgery, I had a hard time financially. I'm currently trying to eat organic food while receiving chemotherapy. Our family budget was greatly affected by the treatment." "I'm come from out of town. I pay a lot of money for transportation, food, and accommodation. Each trip for chemotherapy costs 60 dollars." "Our health insurance usually covers our health expenses. But I paid the difference to have some tests such as magnetic resonance and positron emission tomography done early. I pay 16 dollars out-of-pocket for private examinations every month."
Quality of life	Reducing spending Decreased quality of life	"We limited our expenditure in other areas. This has affected our quality of life. For example, we used to go out for a meal twice a month."
Disease-coping and adaptation	Spiritual and religious coping Visit the turbe and khoja Getting psychological support Spousal, family, and friend support	"I cried secretly for days. I didn't look at my children's faces. I didn't leave the house. Then I tried to pull myself together. I began to think that cancer was something that came from Allah. I said this was God's will; this was my destiny. I told myself that both my troubles and healing came from God. I started to pray. I took my Quran in my hands and read it. Day by day, I began to get used to the diagnosis of cancer and the treatment."

## DISCUSSION

Breast cancer is a disease with very high treatment costs when considered in terms of both healthcare institutions and patients^14^.

In line with the findings of this study, the mean out-of-pocket expenses of the patients in a month were determined to be 165.4±187.7 dollars. It was found that the mean out-of-pocket expenses of the patients for transportation, food, and accommodation during chemotherapy were 11.8±9.2 dollars and the out-of-pocket expenses for the transportation, food, and accommodation of the person accompanying them during chemotherapy treatment were 12.0±13.0 dollars. The mean monthly income loss of the patients who had quit their job due to chemotherapy was 180±139.7 dollars; the mean monthly income loss to their family members for not going to work/inability to work due to the patient's care and treatment was 110.6±96.1 dollars; the mean income loss to the families was 157.3±156.26 dollars.

In other studies, conducted to determine the out-of-pocket expenses of oncology patients, it was reported that these costs varied between 213 and 456 dollars per month^5,22-25^. The out-of-pocket expenses of oncology patients differed in the different studies. It is thought that this may have been affected by the high-, middle-, and low-income levels of the different countries and the level of public financing for cancer care and treatment.

Furthermore, in other relevant studies, it was reported that the out-of-pocket expenses of oncology patients for their cancer care create a significant financial burden on the family budget^5,22^ and the costs are high in the first period of treatment and at the end of life^26^. It was also stated that out-of-pocket expenses affect treatment decisions and these expenses create stress in patients with high out-of-pocket costs compared to those with low or moderate out-of-pocket costs^27^. The inability of individuals to meet the financial burden of the disease was also reported to increase the possibility of delaying treatment, causing the progression of cancer^24,28^. Since out-of-pocket expenses affect the well-being and quality of life of oncology patients, it is important to make interventions aimed at solving this problem^13,15^.

In this study, it was seen that the quality of life and the use of effective coping methods increased as the out-of-pocket expenses of the patients increased. Financial problems due to cancer diagnosis and treatment can lead to a deterioration in the quality of life and psychological well-being of the patient and their family^12^. In one relevant study, it was reported that patients receiving chemotherapy had low quality-of-life scores, the income level of the family was closely associated with the quality of life of breast cancer patients, and a high household income improved the quality of life of breast cancer patients^10^. In another study on the subject, it was emphasized that even insured patients faced the risk of poor quality of life and death as a result of the increasing financial burden of the disease^4,29^. Financial problems related to cancer can negatively affect patients’ access to care, compliance with treatment, and physical health and can thus reduce their quality of life and satisfaction^13^.

During the qualitative interviews, the patients stated that they spent out-of-pocket during their chemotherapy, they had financial difficulties due to the inability of themselves or their family members to work, they spent their savings, they sold their properties, they borrowed money and received help from their close relatives, neighbors, and friends, and they took out bank loans. They emphasized that their out-of-pocket expenses negatively affected their quality of life in particular.

In accordance with the research findings, in some studies on this subject, it was reported that patients used up all their savings/investments^24,25^, borrowed money^24,25,27^, sold their property^24,25^, and changed their lifestyles^25^ in order to pay their medical expenses. Moreover, some patients stated that they only took the most important drugs or reduced the dosage of their medication^25,27^, delayed chemotherapy or clinical appointments^25,27^, did not have control procedures and tests^25^, and had to choose between food and medicine and their out-of-pocket expenses were generally for accommodation and transportation^27^.

In the current study, the patients stated that they received psychological and psychiatric support to cope with and adapt to their disease, their orientation toward the spiritual increased, and they resorted to religious coping methods (rituals to remove bad luck, a pilgrimage to a turbe [an Ottoman royal tomb], visiting a khoja [Muslim religious leader/holy man]).

It was emphasized that it is extremely important for women with breast cancer to be able to cope with the stress resulting from the disease and to use appropriate coping methods^30,31^. A low level of income may also limit patients’ access to all kinds of care and treatments^12,24^. For this reason, it is thought that identifying low-income patients and their relatives and ensuring they have access to health services are extremely important in increasing their quality of life^32^.

In order to combat the financial burden of the out-of-pocket health expenses of breast cancer patients, develop appropriate policies regarding this issue, and obtain reliable, valid, and comparable health-related information at both national and international levels, there is a need to shine a light on these expenses and their effect on the quality of life of and disease coping. It is thus important to evaluate out-of-pocket expenses from the perspective of cancer patients.

To mitigate the negative effects of out-of-pocket expenses on patient health, providing social and economic support could help improve quality of life. Additionally, psychosocial support to assist with coping and financial assistance to relieve economic burdens are recommended, and these interventions could be implemented in clinical settings.

### Strengths and limitations

This research has some limitations. A limitation of this research is that the study was carried out in only one hospital. Until comparable studies are conducted on breast cancer patients with different socio-cultural characteristics, the findings of this study cannot be generalized to all breast cancer patients. The strength of this study is that the population of this study consisted of only breast cancer patients and was homogeneous.

## CONCLUSION

In this study, it was determined that the patients’ quality of life and the use of effective coping methods increased as their out-of-pocket expenses increased. As a result of the qualitative interviews held with the patients, it can be said that the out-of-pocket expenses of the breast cancer patients during the chemotherapy process affected their quality of life, disease coping, and adaptation to their situation.

Patients and their relatives who experience financial difficulties should be directed to institutions that provide social support and financial aid. Patients and their relatives should be informed about which expenses are covered by the healthcare system. Individualized nursing care that will improve patients’ ability to cope with the changes in their lives should be planned and implemented. The quality of life of patients should be evaluated at periodic intervals. Oncology patients should be supported in the process of coping with and adapting to cancer. Psychological counseling and guidance services should be provided to patients in need.
